# Intraoperative MRI in pediatric brain tumor surgery: Optimizing surgical decision-making and extent of resection

**DOI:** 10.1007/s00381-026-07456-w

**Published:** 2026-09-17

**Authors:** Jennifer To, Timothy Müller, Oliver Bozinov, Luca Regli, Niklaus Krayenbühl, Julia Velz

**Affiliations:** 1 Department of Neurosurgery, Clinical Neuroscience Center, University Hospital Zurich, Frauenklinikstrasse 10, 8091 Zurich, Switzerland; 2 University of Zurich, Zurich, Switzerland; 3 Department of Pediatric Neurosurgery, University Children’s Hospital Zurich, Lenggstrasse 30, 8008 Zurich, Switzerland; 4 Department of Neurosurgery, Kantonsspital St. Gallen, Rorschacherstrasse 95, 9007 St. Gallen, Switzerland

**Keywords:** Pediactric brain tumor, Intraoperative MRI, Brain tumor surgery, Extent of resection, Secondary resection

## Abstract

**Objective:**

To assess the impact of intraoperative MRI (ioMRI) on the extent of resection (EoR) in pediatric brain tumor surgery and evaluate the benefits and limitations of its routine implementation.

**Methods:**

Observational single-center study, including prospectively collected data of 118 ioMRI-guided intracranial tumor resections in 108 children that were performed at the University Hospital Zurich between May 2013 and October 2022. EoR was graded as gross total (GTR; no residual tumor), sub-total (STR; > 90% tumor resection), and partial resection (PR; < 90% tumor resection).

**Results:**

A total of 118 ioMRI-guided interventions were performed in 108 individual children aged ≤ 18 years. The intended EoR determined pre-operatively was GTR in 81 cases (68.6%), STR in 24 (20.3%), and PR in 13 (11.0%). IoMRI confirmed the intended EoR achieved in 78/118 procedures (66.1%), demonstrating GTR in 48 cases (40.7%), STR in 50 (42.4%), and PR in 20 (16.9%). In 48 cases (40.7%), ioMRI findings prompted further intraoperative evaluation; additional tumor resection was performed in 45 cases (93.8%), resulting in improved EoR. The final EoR distribution after ioMRI-guidance was 80 GTR (67.8%), 25 STR (21.2%), and 13 PR (11.0%), resulting in a rate of achieving the intended EoR in 99.2% of cases (117/118). Surgical site infections (SSI) occurred in 4 cases (3.4%).

**Conclusions:**

This study highlights the substantial impact of ioMRI in pediatric brain tumor surgery, demonstrating a high rate of surgical site reopening for residual tumor resection, which substantially improved the EoR and minimized the need for a second operation – without increasing SSI incidence.

## Introduction

Brain tumors are the most common solid pediatric cancer and the leading cause of cancer related mortality in the pediatric population [11, 19, 27]. Besides the histopathological entity, tumor location and age at presentation, the extent of resection (EoR) is often a relevant optimizable prognostic factor [8, 21, 32]. Consequently, a substantial proportion of children require a second surgery because postoperative MRI reveals that the intended EoR has not been achieved [3, 6, 25].

Over the last two decades intraoperative MRI (ioMRI), allowing for intraoperative assessments of the achieved EoR and updating neuronavigation, has become a beneficial and established tool in many different neurosurgical procedures, including the management of adult neuro-oncological patients by maximizing safe resection and thus improving prognosis. Despite its wide implementation in adult patients, the use of ioMRI in the pediatric population is still very limited. While its potential has been increasingly recognized [2, 3, 5, 6, 12–14, 17] and more recent studies have demonstrated the utility and safeness of this tool in the pediatric population [7, 9, 10, 16, 20, 29, 30], its impact on intraoperative surgical decision-making and extent of resection (EoR) remains insufficiently studied. Furthermore, most published studies have focused exclusively on gliomas, predominantly low-grade gliomas [9, 12, 20, 30]. Given the relatively high initial acquisition and maintenance cost of an ioMRI system in a Neurosurgical Department, as well as the concerns about prolonged operation times and the associated risk of increased surgical site infections (SSI), ioMRI is currently only used in a minority of Neurosurgical Departments for pediatric brain tumor surgery.

The aim of this observational study is to evaluate the impact and utility of ioMRI in pediatric brain tumor surgery by assessing its effect on the EoR, the number of additional resection secondary to ioMRI and the rate of SSI in our neurosurgical institution, as well as the discussion of further benefits and limitations to provide more data for establishing its future use.

## Methods

### Patient selection

We prospectively collected data from the patient registry of the Department of Neurosurgery at the University Hospital Zurich [23]. We screened all patients aged ≤ 18 who underwent surgical ioMRI-guided intracranial tumor resection at the Department of Neurosurgery at the University Hospital of Zurich in Switzerland between May 2013 and October 2022. All surgeries were performed by board-certified neurosurgeons with over 15 years of experience in treating pediatric and adult brain tumors. In our institution, intraoperative ultrasound is routinely used during all intracranial tumor resections. In addition, intraoperative neurophysiological monitoring is routinely employed for tumors located within or adjacent to eloquent neural structures, including the motor cortex, brainstem, and cranial nerves, to facilitate safe maximal tumor resection.

### Data acquisition

The prospectively collected medical records of all patients were reviewed for baseline patient and tumor characteristics, including age at surgery, sex, tumor location, histopathological diagnosis and molecular tumor classification. Procedure-specific parameters, including whether the procedure represented the initial tumor resection and the total operative time, were also recorded.

The preoperatively intended EoR was determined based on the surgical reports and the multidisciplinary neuro-oncological tumor board discussions involving neurosurgeons, neuroradiologists, and pediatric oncologists. The intended EoR was defined by the tumor board prior to surgery, primarily based on tumor type, anatomical location, and anticipated surgical feasibility.

The achieved EoR was assessed using preoperative and intraoperative MRI datasets, together with the corresponding radiological reports generated by senior neuroradiologists. The EoR after the initial resection was determined by comparing preoperative and intraoperative MRI findings. In patients undergoing secondary resection prompted by intraoperative MRI, postoperative MRI was additionally reviewed to determine the final EoR. In cases without secondary resection, the intraoperative MRI findings were considered representative of the final EoR, as routine postoperative MRI was not performed. All EoR assessments were conducted by experienced neuroradiologists using a standardized, semi-automated volumetric analysis tool integrated into the institutional PACS system.

The occurrence of surgical site infections (SSI) and early reoperation was also additionally recorded.

### Two-room intraoperative MRI system

Our institution uses a 3 T intraoperative magnetic resonance imaging (ioMRI) suite based on a two-room concept, consisting of a standard operating room directly connected to a MRI scanner room (MAGNETOM Skyra, Siemens Healthineers, Erlangen, Germany) via a sliding double door. The MRI scanner remains fixed while the patient is transferred between the operating room and the scanner room. Outside of ioMRI-guided procedures the MRI system is available for routine clinical imaging and the operating room can be independently used.

In our institution ioMRI is routinely used for all pediatric brain tumor resections. Patients requiring a sitting or park-bench position are excluded from ioMRI, as patient transfer to the MRI scanner requires positioning in either the supine or prone position.

All patients undergo standard preoperative MRI safety screening according to the institutional protocol. Surgical procedures are performed using MRI-compatible anesthesia equipment and a non-ferromagnetic cranial fixation system (DORO LUCENT® ioMRI Cranial Stabilization System, Getinge, Rastatt, Germany).

Further details and illustrative images of our two-room ioMRI setup and the standardized patient transfer procedure and safety protocol have been previously described [28].

### Intraoperative MRI workflow and surgical decision-making

Before each procedure, a modified WHO “Safe Surgery Checklist” adapted for ioMRI-guided surgery is completed at predefined time points by the neurosurgeon, neuroanesthesiologist, and operating room nursing staff [28].

During surgery, intraoperative MRI is performed once the operating surgeon considers the planned EoR to have been achieved.

After meticulous hemostasis, the dura is temporarily approximated without definitive watertight closure, and a hemostatic sponge is placed over the dural opening to minimize blood entering the resection cavity during image acquisition. The skin is temporarily approximated, covered with sterile gauze dressings, and sealed with a sterile surgical drape to maintain sterility throughout patient transfer and imaging. The patient remains fixed in the MRI-compatible head holder, and the lower and upper head coils are installed before transfer to the adjacent MRI scanner room following the standardized Zurich ioMRI transport protocol and safety checklist, as previously described by Stienen et al. [28].

Following image acquisition, the patient is returned to the operating room and the temporary wound closure and sterile dressings are removed. The ioMRI images are reviewed by the operating surgeon together with an attending neuroradiologist.

If ioMRI confirms the intended extent of resection, definitive watertight dural closure, replacement of the bone flap, and final wound closure are performed. If residual tumor considered safely resectable is identified, neuronavigation is updated using the ioMRI dataset, the operative field is reopened, and tumor resection is continued until the intended extent of resection is achieved or further resection is considered unsafe.

### Statistical analysis

Baseline characteristics and clinical data were summarised and analyzed using standard descriptive statistics and are given as absolute numbers and percentages.

## Results

Between May 2013 and October 2022, 123 ioMRI-guided intracranial tumor resections were planned for a total of 111 children aged ≤ 18. Among these, 118 ioMRI-guided interventions performed in 108 individual children were included in the final analysis. Five out of 123 interventions were excluded, as ioMRI was planned for them, but not performed: two due to technical issues, one due to prolonged operation time, one deemed unnecessary based on the surgeon's judgment, and one due to intraoperative surgical complications.

### Patient demographics and basic data of performed ioMRI interventions

108 children aged 18 years or younger underwent ioMRI-guided brain tumor resection surgery and were included in the final anaylsis (*n* = 49 females, 45.4%). The median age at first ioMRI-guided intervention was 8.6 years (range 1.2–18 years).

The most frequent brain tumors were Medulloblastomas (21/108, 19.4%), Pilocystic Astrocytomas (19/108, 17.6%), Gliomas (18/108, 16.7%) and Ependymomas (13/108, 12.0%). The majority of the tumors were located in the IV ventricle (27/108, 25.0%). Other tumor locations included the hemispheres (20/108, 18.5%), cerebellum (18/108, 16.7%), and basal ganglia and thalamus (18/108, 16.7%). Patient demographics, detailed tumor locations and histopathological diagnoses are described in Table 1.

**Table 1:** Patient demographics

**Number of Children**	108	
**Female**	49	(45.4%)
**Median age at first intervention**	8.6 (range 1.2–18)	
**Tumor location**		
IV Ventricle	27	(25.0%)
Hemispheres	20	(18.5%)
Basal ganglia and Thalamus	18	(16.7%)
Cerebellum	18	(16.7%)
Brainstem	5	(4.6%)
Pituitary gland	5	(4.6%)
Posterior cranial fossa	4	(3.7%)
Central region	3	(2.8%)
Lateral and III ventricles	3	(2.8%)
Middle cranial fossa	3	(2.8%)
Anterior cranial fossa	2	(1.9%)
**Histology**		
Medulloblastoma	21	(19.4%)
Other	21	(19.4%)
Pilocystic Astrocytoma	19	(17.6%)
Glioma	18	(16.7%)
Ependymoma	13	(12.0%)
Ganglioglioma	6	(5.6%)
Craniopharyngeoma	5	(4.6%)
Pituitary adenoma	3	(2.8%)
ATRT	2	(1.9%)

*ATRT* Atypical teratoid/rhabdoid tumor

A total of 118 ioMRI-guided interventions were performed in our cohort of 108 children. 73 interventions (61.9%) were first operations. The median total operation room time, defined as the time from skin incision to definitive wound closure including the intraoperative MRI acquisition, was 336.5 min (range 140–670 min). Surgical site infections occurred in 4 cases (3.4%).

Consistent with the patient demographics, the most common brain tumor in all the ioMRI-guided interventions was Medulloblastoma (23/118, 19.5%) followed by the Pilocystic Astrocytoma (21, 17.8%), Glioma (20, 16.9%) and Ependymoma (13, 11.0%). 28 of all 118 interventions were performed in the IV ventricle (23.7%), 23 in the hemispheres (19.5%), 21 in the basal ganglia and thalamus (17.8%) and 20 in the cerebellum (16.9%). See Table 2 for more details.

**Table 2:** Basic data of performed surgical ioMRI guided interventions

**Number of surgical ioMRI guided interventions**	118	
**First operation**	73	(61.9%)
**Media operation room time [min]**	336.5 (range 140–670)	
**Surgical site infections**	4	(3.4%)
**Tumor location**		
IV Ventricle	28	(23.7%)
Hemispheres	23	(19.5%)
Basal ganglia and Thalamus	21	(17.8%)
Cerebellum	20	(16.9%)
Brainstem	6	(5.1%)
Pituitary gland	5	(4.2%)
Posterior cranial fossa	4	(3.4%)
Central region	3	(2.5%)
Lateral and III ventricels	3	(2.5%)
Middle cranial fossa	3	(2.5%)
Anterior cranial fossa	2	(1.7%)
**Histology**		
Medulloblastoma	23	(19.5%)
Other	23	(19.5%)
Pilocystic Astrocytoma	21	(17.8%)
Glioma	20	(16.9%)
Ependymoma	13	(11.0%)
Craniopharyngeoma	7	(5.9%)
Ganglioglioma	6	(5.1%)
Pituitary adenoma	3	(2.5%)
ATRT	2	(1.7%)

*ATRT* Atypical teratoid/rhabdoid tumor, *ioMRI* Intraoperative magnetic resonance imaging

### IoMRI assessment and additional resection

GTR was intended in 81 out of 118 cases (68.6%). Among these, ioMRI confirmed that GTR had been achieved in 48 cases (59.3%). In the remaining 33 cases (40.7%), ioMRI suggested possible residual tumor, prompting further microscopic exploration with updated intraoperative neuronavigation. Additional resection was performed in 30 of these cases (37.0%), improving the extent of resection (EoR) to GTR in 29 out of 30 cases. In the remaining case, only additional debulking was achieved without reaching GTR, resulting in an overall GTR rate of 98.8% (80/81 cases). In the remaining 3 of the 33 cases where ioMRI indicated possible residual tumor, microscopic exploration confirmed that the suspected residual lesion represented normal anatomical structures (e.g., a blood vessel or choroid plexus) rather than residual tumor. Consequently, no further resection was performed, as complete gross total resection (GTR) had already been achieved.

In 24 of 118 cases (20.3%), the pre-operatively determined intended extent of resection was subtotal resection (STR, > 90%). IoMRI confirmed the planned STR achieved STR in 17 cases (70.8%). Additional resection was performed in 12 cases (50%), including all 7 cases in which ioMRI indicated that only a partial resection (PR) had been achieved, as well as 5 cases in which the intended STR had already been achieved but ioMRI demonstrated that further safe debulking was feasible. Ultimately, the intended STR was achieved in all 24 cases (100%).

In 13/118 (11.0%) cases, the intended extent of resection was PR, which was achieved in all 13 cases. In 3 of these cases (23.1%), ioMRI demonstrated that safe additional debulking was feasible, and further tumor resection was therefore performed while maintaining the planned EoR.

Overall, additional tumor resection following ioMRI was performed in 45 of 118 cases (38.1%), resulting in an improved extent of resection (EoR). Based on the ioMRI findings, further microscopic exploration with updated intraoperative neuronavigation was undertaken in 48 of 118 cases (40.7%). Detailed results are summarized in Table 3.

**Table 3:** ioMRI assessment and rates of additional tumor resection after ioMRI

Intended EoR	*n*	ioMRI indicated		Cases undergoing additional exploration	Cases with improved EoR
		Intended EoR achieved	Intended EoR not achieved		
GTR	81 (68.6%)	48 (59.3%)	33	33 (40.7%)	30 (37.0%)
STR	24 (20.3%)	17 (70.8%)	7	12 (50%)	12 (50%)
PR	13 (11.0%)	13 (100%)	0	3 (23.1%)	3 (23.1%)
total	118 (100%)	78 (66.1%)	40	48 (40.7%)	45 (38.1%)

*EoR* Extent of resection, *GTR* Gross total resection, *ioMRI* intraoperative Magnetic resonance imaging, *PR* Partial resection (< 90%), *STR* subtotal Resection (> 90%)

### Surgical outcome and extent of resection

**Fig. 1:**
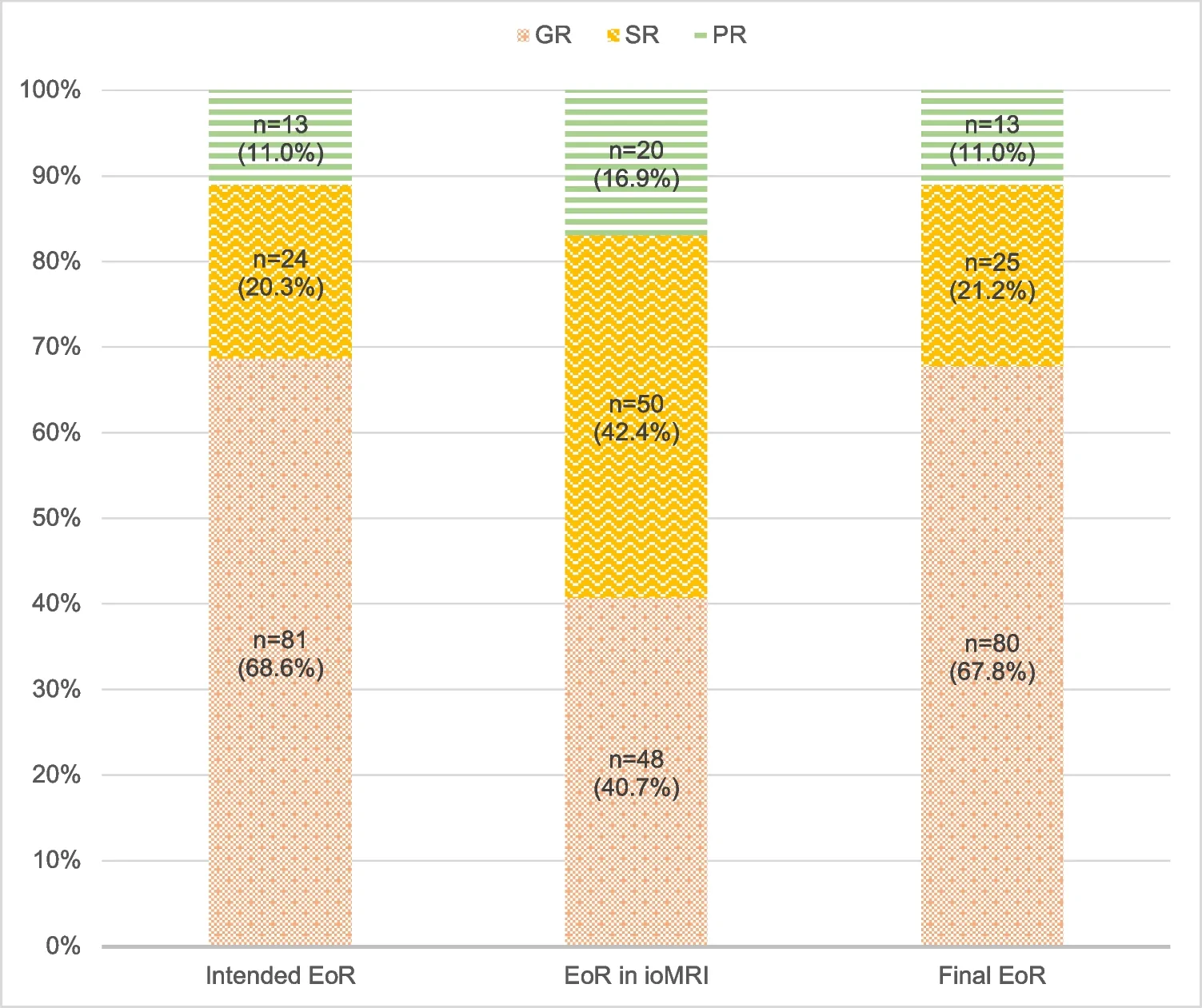
Distribution of the preoperatively Intended, Intraoperative ioMRI-assessed, and Final EoR in 118 pediatric brain tumor surgeries. Gross total resection (GTR, orange/dotted), Subtotal resection (STR, yellow/waves), and Partial resection (PR, green/striped) are shown. IoMRI revealed a lower rate of GTR and higher STR and PR compared to the Intended EoR. Following ioMRI-guided additional resection (performed in 45/118 cases) the Final EoR closely approximated the preoperatively Intended EoR

In summary, the preoperatively intended extent of resection (EoR) was gross total resection (GTR) in 81 cases (68.6%), subtotal resection (STR) in 24 (20.3%), and partial resection (PR) in 13 (11.0%). Following the initial resection, ioMRI demonstrated GTR in 48 cases (40.7%), STR in 50 (42.4%), and PR in 20 (16.9%), indicating that the planned EoR had been achieved in 78 of 118 cases (66.1%). Based on the ioMRI findings, additional tumor resection was performed in 45/118 cases (38.1%). This resulted in a final EoR of GTR in 80 (67.8%), STR in 25 (21.2%), and PR in 13 cases (11.0%), corresponding to achievement of the intended EoR in 117 of 118 cases (99.2%) (Fig. 1). Notably, in 8 of the 45 cases undergoing secondary resection, the planned EoR category had already been achieved after the initial resection—however, ioMRI demonstrated that additional safe tumor debulking was feasible, allowing further reduction of the residual tumor burden without changing the intended EoR category.

### Case illustrations

**Fig. 2:**
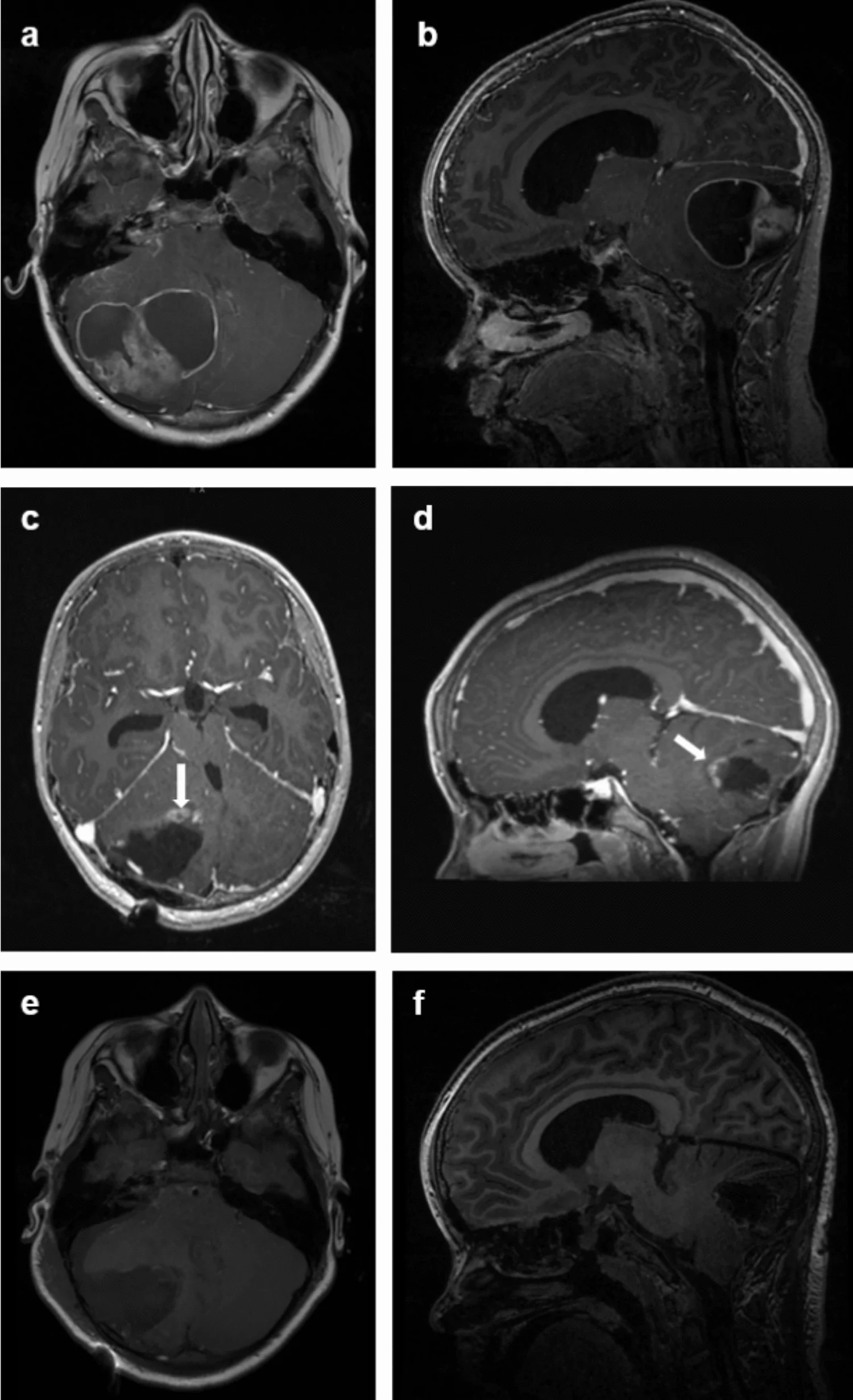
A 12-year-old patient presented with headaches, vertigo, and morning vomiting persisting over several weeks. Preoperative MRI (contrast-enhanced T1-weighted images, axial [**a**] and sagittal [**b**]) demonstrated a cystic mass in the right cerebellum with a solid, contrast-enhancing component, suggestive of a pilocytic astrocytoma. Gross total resection (GTR) was the intended extent of resection (EoR). Intraoperative MRI (**c**, **d**) revealed persistent contrast enhancement at the anterior–superior margin of the resection cavity (arrow), indicating residual tumor and demonstrating that only a subtotal resection (STR) had been achieved. Additional tumor resection was performed. Postoperative MRI (**e**, **f**), obtained the day after surgery, confirmed complete tumor removal, resulting in the final EoR of GTR

**Fig. 3:**
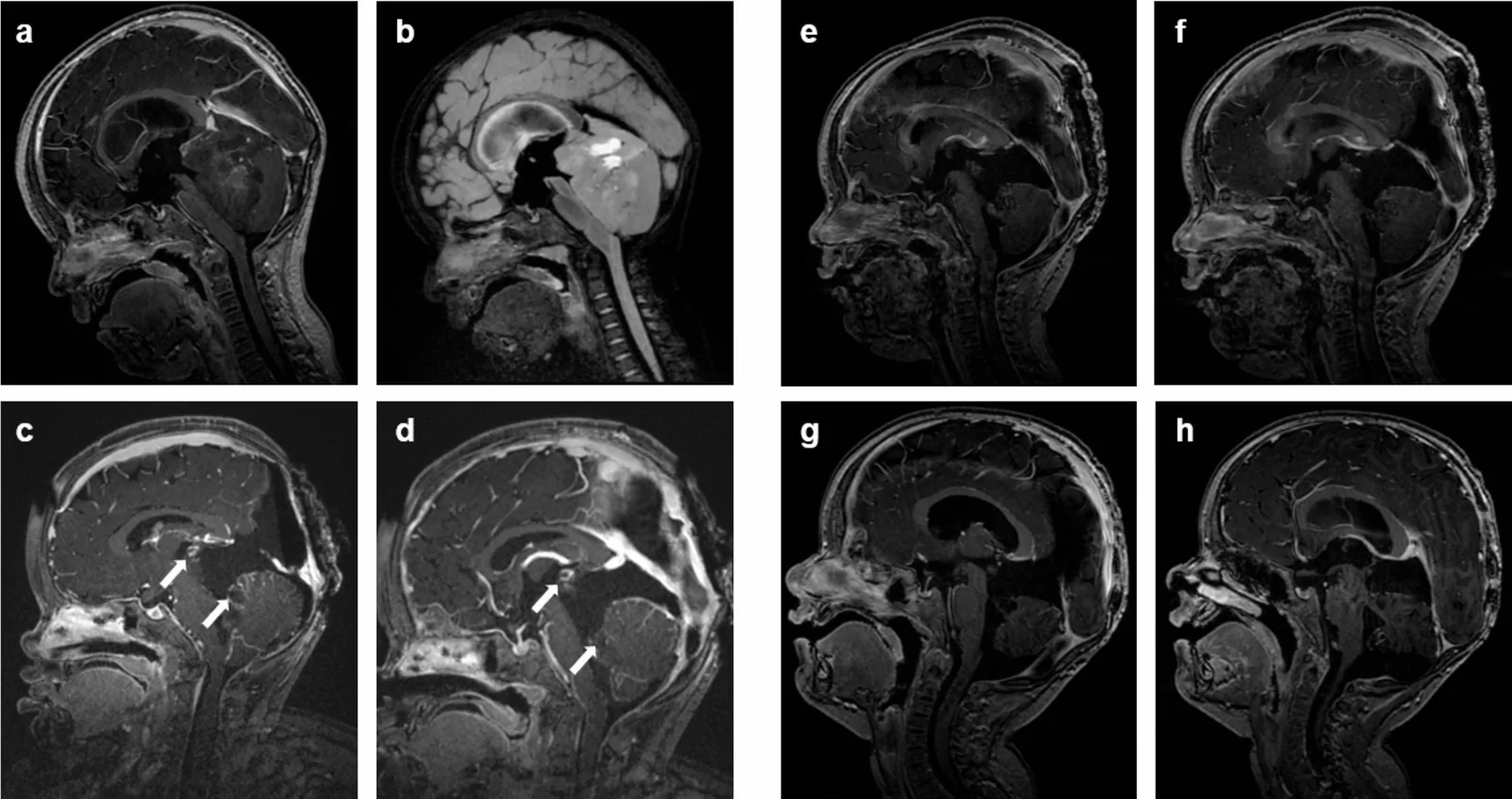
A 1.5-year-old patient presented with an acute onset of ataxia, recurrent falls, and vomiting. Preoperative MRI (T1-weighted with contrast sagittal [**a**], FLAIR sagittal [**b**]) revealed a large mass in the cisterna laminae quadrigeminae. Based on the imaging findings, an ATRT was suspected, and the tumor board recommended gross total resection (GTR) as the surgical goal. Intraoperative MRI (**c**, **d**) confirmed that the majority of the tumor had been removed but demonstrated multiple small residual tumor nodules (arrow). Additional tumor resection was therefore performed. Postoperative MRI (**e**, **f**), obtained the following day, confirmed complete tumor removal resulting in a final extent of resection (EoR) of GTR. The patient subsequently received adjuvant proton radiotherapy according to the EU-RHAB protocol and chemotherapy. Follow-up MRI at 4 months postoperatively showed no evidence of tumor recurrence (**g**, **h**)

**Fig. 4:**
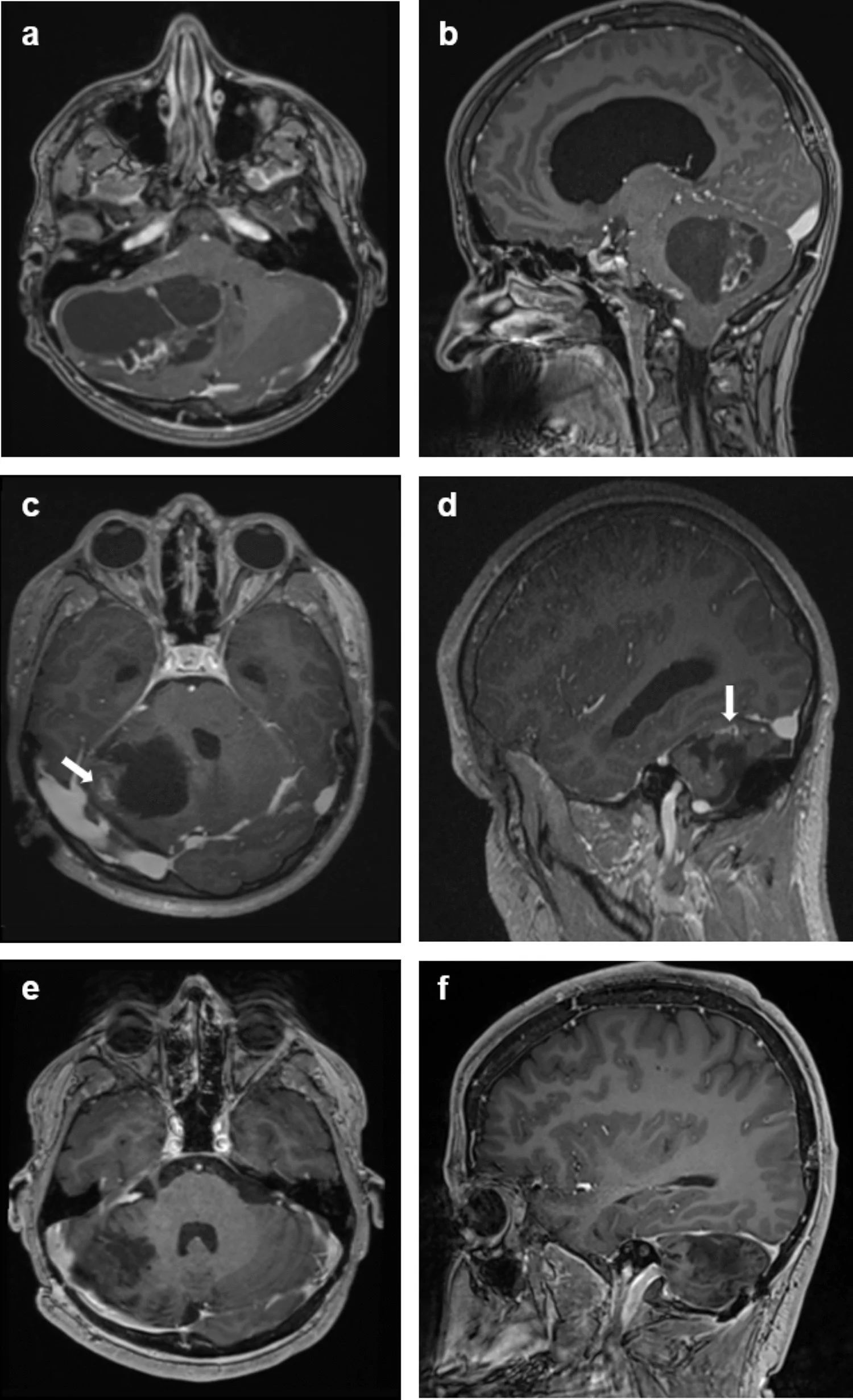
A 17-year-old patient presented with several months of headaches and vomiting occurring on an empty stomach. Preoperative MRI (T1-weighted axial [**a**] and sagittal [**b**]) demonstrated a cystic mass in the right cerebellum, suggestive of a pilocytic astrocytoma. Gross total resection (GTR) as the intended extent of resection (EoR). Intraoperative MRI (**c**, **d**) demonstrated residual contrast-enhancing tumor residues at the superolateral margin of the resection cavity (arrow), indicating that only a subtotal resection (STR) had been achieved. Additional tumor resection was therefore performed. Postoperative MRI (**e**, **f**), obtained the following day, confirmed complete tumor removal, resulting in a final EoR of GTR

Figures 2, 3, 4 illustrate representative cases in which ioMRI identified residual tumor after the initial resection, enabling additional resection during the same procedure and resulting in achievement of the intended EoR. Each figure includes the preoperative, intraoperative and early postoperative MRI examinations for each case.

## Discussion

The main objective of this study was to assess the impact of ioMRI on pediatric brain tumor surgery, specifically its influence on intraoperative surgical decision-making and its effect on achieving the intended EoR.

This series shows a marked improvement in the EoR, with a final rate of 99.2% (117/118; 95% CI 0.975–1.00, *p* < 0.05) after ioMRI, compared to 66.1% (78/118; 95% CI 57.6–74.6) before ioMRI, aligning with previous pediatric studies [2, 10, 12–14, 17, 20, 29, 30]. Additional tumor resection was performed in 38.1% (45/118) of cases based on ioMRI findings. These resections addressed residual tumor, that was either part of the intended EoR but not initially achieved, or lesions that had been considered unresectable preoperatively. The latter scenario, in which ioMRI enabled achieving a greater EoR than initially intended, occurred in 7% of all resections (8/118). In 5 of these 8 cases the pre-operatively determined surgical aim was STR, and none developed neurological deficits. In the remaining 3 cases where PR was the surgical aim 2 patients presented postoperative complications. These complications (focal seizure and acute hydrocephalus) cannot be directly attributed to the additional resection. This highlights ioMRI's value, especially for tumors in highly eloquent areas, where GTR is medically not or rarely indicated, but every additional debulking still valuable, similarly shown in Millward et al. [18] for optic pathway and hypothalamic gliomas. For example, in craniopharyngeomas, ioMRI allows intraoperative assessment of cyst opening. In the mentioned 45 cases, additional tumor removal prevented the need for early reoperation, thereby reducing the risks associated with repeat surgery and anesthesia, and decreasing the total rate of early reoperation in our cohort to 0% (0/118 cases).

While additional intraoperative reassessment based on ioMRI occurred in 40.7% of cases, it was associated with a significant improvement in the achieved EoR and a higher rate of GTR. These findings highlight the clinical relevance of ioMRI in optimizing intraoperative decision-making and are consistent with previous reports in pediatric [24] and adult cohorts [15, 31, 34].

Despite concerns about increased risk of surgical site infections (SSI) due to imaging with an open wound in a sterile field and extended surgery time, no significant difference in SSI rates was found in our 118 ioMRI-guided surgeries compared to non-ioMRI pediatric brain tumor resections reported in the literature [26]. Previous studies confirm similar findings [12, 20, 29, 30]. Thus, our data supports ioMRI as a safe and beneficial tool in pediatric brain tumor surgery by maximizing safe resection.

Even when no additional resection is performed, ioMRI provides a reliable postoperative baseline, reducing the need for postoperative MRI under sedation, which remains routine practice in many pediatric neurosurgical centers [1]. Furthermore, ioMRI enables intraoperative updating of neuronavigation to compensate for brain shift and anatomical changes following tumor resection. While our two-room ioMRI setup requires patient positioning in either the supine or prone position and therefore precludes procedures performed in the sitting or park-bench position, it does not otherwise interfere with the surgical workflow, consistent with the experience reported by Tejada et al. and Kubben et al. [14, 29].

The main limitations of implementing ioMRI in neurosurgical centers are the high acquisition and maintenance cost, the logistical requirements for a two-room setup and the need for MRI-compatible equipment [22]. In addition, ioMRI prolongs operative time and thereby may theoretically increase the risk of perioperative complications. However, in institutions with a dedicated two-room setup, the MRI scanner and operating room can be used independently outside ioMRI procedures, which may partially offset these costs. Given the prognostic importance of maximizing safe resection in pediatric brain tumors [2, 3, 6, 9, 25], these challenges must be balanced against the potential clinical benefits of achieving a greater extent of resection. The potential advantages of ioMRI may be particularly relevant in re-operations, where surgical complexity is increased – this warrants further investigation in future studies.

### Limitations of this study

Despite prospective data collection, there were some gaps and missing information, particularly in earlier cases, as the completeness and standardization of the registry improved over time. Additionally, certain factors may have influenced surgical decisions during the procedure. For example, the availability of ioMRI as a planned control step can lead to its early use – before the surgeon actually believes to have fully completed the intended resection. In such cases, the ioMRI may be performed even if the surgeon would have continued resecting without it.

Furthermore, the availability of ioMRI tends to lower the threshold for reopening the surgical site intraoperatively. Surgeons are often more inclined to investigate and remove small residual tumor tissue when it is detected during surgery, whereas such findings might be left untreated if only identified on postoperative imaging—when reoperation would be required. These dynamics may slightly influence the observed rates of achieving the preoperatively intended EoR at the time of ioMRI and the rate of intraoperative reopening. However, we believe these effects are inherent to the practical use of ioMRI and reflect real-world surgical decision-making.

### Strenghts of this study

Unlike most studies on ioMRI in pediatric brain tumor surgery, which focus on low-grade gliomas [9, 12, 20, 30] and its effect on GTR rates [3, 4, 20, 30, 33], this study analyzed ioMRI's impact on all intracranial tumor resections at our institution, including a broad range of tumor entities. Since GTR is not an appropriate surgical goal for all tumors, we compared the final EoR achieved after ioMRI with the preoperatively intended EoR for each case. This within-procedure comparison allowed assessment of the added value of ioMRI by evaluating the change from the initially achieved EoR before ioMRI (surrogate for without ioMRI) to the final EoR after ioMRI, thereby reducing the limitations associated with separate control and treatment cohorts.

Lastly, a key strength of this study is the sizable cohort, which includes a significant number of pediatric patients, especially considering the still relatively limited use of ioMRI in children's brain tumor surgery.

## Conclusion

This study shows that the use of ioMRI was associated with increased rates of achieving the intended EoR in pediatric brain tumor surgery. IoMRI-guidance enabled an improvement of the EoR in more than one-third of the cases without an increase in SSI rates. Given the prognostic relevance of EoR and the additional technical advantages of ioMRI, including intraoperative neuronavigation updates and acquisition of an early postoperative baseline scan, ioMRIs represent a valuable adjunct in pediatric neuro-oncological surgery. Our findings demonstrate that ioMRI can be applied safely and effectively across tumor entities, supporting its broader implementation to optimize resection outcomes under appropriate logistical and financial conditions.

## Data Availability

The data can be made available upon reasonable request to the corresponding author.

## Abbreviations

ATRT: Atypical teratoid/rhabdoid tumor
EoR: Extent of Resection
GTR: Gross Total Resection
ioMRI: Intraoperative magnetic resonance imaging
PR (< 90%): Partial Resection (< 90%)
SSI: Surgical site infections
STR (> 90%): Subtotal Resection (> 90%)
